# ‘To be Informed and Involved’: Women's insights on optimising childbirth care in Lithuania

**DOI:** 10.1111/hex.13754

**Published:** 2023-06-06

**Authors:** Giedrė Širvinskienė, Švitrigailė Grincevičienė, Rasa Pranskevičiūtė‐Amoson, Milda Kukulskienė, Soo Downe

**Affiliations:** ^1^ Department of Health Psychology, Faculty of Public Health Lithuanian University of Health Sciences Kaunas Lithuania; ^2^ Health Research Institute, Faculty of Public Health Lithuanian University of Health Sciences Kaunas Lithuania; ^3^ Department of Biothermodynamics and Drug Design, Institute of Biotechnology, Life Science Centre Vilnius University Vilnius Lithuania; ^4^ Institute of Asian and Transcultural Studies Vilnius University Vilnius Lithuania; ^5^ ReaCH Group, THRIVE Centre University of Central Lancaster Preston UK

**Keywords:** Babies Born Better survey, childbirth, Lithuania, maternity, qualitative research, women's experiences and views

## Abstract

**Introduction:**

The user expectations and experiences of healthcare services are acknowledged as components of the quality of healthcare evaluations. The aim of the study is to analyse women's experiences and views on childbirth care in Lithuania.

**Methods:**

The study used the Babies Born Better (B3) online survey as the data collection instrument. The B3 is an ongoing longitudinal international project, examining the experiences of intrapartum care and developed as part of EU‐funded COST Actions (IS0907 and IS1405). Responses to open‐ended questions about (1) the best things about the care and (2) things in childbirth care worth changing are included in the current analysis. The participants are 373 women who had given birth within 5 years in Lithuania. A deductive coding framework established by the literature review was used to analyse the qualitative data. The framework involves three main categories: (1) the service, (2) the emotional experience and (3) the individually experienced care, each further divided into subcategories.

**Results:**

Reflecting the experience and views regarding the *service* at birthplace women wished empowerment, support for their autonomy and to be actively involved in decisions, the need for privacy, information and counselling, especially about breastfeeding. In terms of *emotional experience*, women highlighted the importance of comprehensibility/feeling of safety, positive manageability of various situations and possibilities for bonding with the newborn. *Individually experienced care* was described by feedback on specific characteristics of care providers, such as competence, personality traits, time/availability and encouragement of esteem in women in childbirth. The possibilities of homebirth were also discussed. The findings reflected salutogenic principles.

**Key Conclusions:**

The findings suggest that the Lithuanian healthcare system is in a transition from paternalistic attitude‐based practices to a shift towards patient‐oriented care. Implementation of the improvements suggested for women in childbirth care in Lithuania would require some additional services, improved emotional and intrapersonal aspects of care and a more active role for women.

**Patient/Public Contribution:**

Patients and the public contributed to this study by spreading information about surveys and research findings through their involvement in service user groups that have an interest in maternity care. Members of the patients' groups and the public were involved in the discussion of the results.

## 1 INTRODUCTION

Around the world, there is a shift in the focus of health care, from survival only, to also emphasising patient‐centred care, health improvement and subjective patient wellbeing. The user expectations and experiences of healthcare services are acknowledged as components of quality of care evaluations^1^ and as a reflection of interactions between patients and the health system.^2^

The World Health Organisation vision of quality of care for pregnant women and newborns includes a women‐centred component, taking into account the preferences and aspirations of individual service users,^3^ with criteria for quality evaluation involving women's experience of effective communication, respect and preservation of dignity and emotional support.^4^ The current guidelines recognise that the notion of a positive experience is as critical as good clinical outcomes for babies,^5^, ^6^ based on a systematic review of what matters to women during childbirth.^7^

Childbirth is a critical moment in the transition to motherhood. This transformation can be positive or stressful, as a labouring woman is simultaneously in a powerful and potentially vulnerable state, both clinically and emotionally.^8^ As a complex and multidimensional experience of care, it has an impact on the future wellbeing of both mother and child.^9^, ^10^, ^11^ A negative mother's perception of birth care experience is associated with postpartum anxiety and depression.^11^ Postpartum depression has been linked to a reduced capacity to perceive an infant's emotions and respond adequately.^12^, ^13^ Women's accounts of their birth care experience could be a useful early indicator for later wellbeing or for potential psychological adversity. Supporting women to make informed decisions by providing information about birth setting options and variations is a critical patient‐centred strategy.^14^

There is strong evidence that if a woman feels she has a sense of control in labour and is well supported, she is more likely to experience her childbirth care as positive.^15^, ^16^, ^17^ However, while there is a large body of literature on women's experiences of childbirth care from North America, northern Europe and Australasia, there is far less research on this topic from Eastern European countries, where maternal engagement in maternity care choices and practices appears to be less prominent. Few studies have analysed women's attitudes towards maternity care in Lithuania. One of them revealed that more than half of the women were satisfied with fulfilment of their expectations during childbirth and postpartum care expectations.^18^

The aim of the study was to analyse women's experiences and views on childbirth care in Lithuania. Specific goals were to explore women's reports of positive aspects of care by birthplace and to identify areas in which women would like to see improvements in the future.

## 2 MATERIALS AND METHODS

### 2.1 Procedure and sample

The study used the Babies Born Better (B3) survey as the data collection instrument. The B3 survey is an ongoing longitudinal international project, examining the views and experiences of childbirth care. It aims to highlight areas of good practice in childbirth care around the world, geo mapped to place of birth, so that all services can learn and apply what works well in those services rated most highly by respondents. The survey was developed and refined as part of two EU‐funded COST Actions (IS0907 and IS1405). These Actions were designed to advance scientific knowledge about ways of improving maternity care provision and outcomes for mothers, babies and families across Europe, by understanding what works, for whom and in what circumstances. The survey is based on a salutogenic perspective, which concentrates on health promotion and understanding what goes well, while including negative outcomes and experiences, in a health continuum.^19^ The key concepts of salutogenesis are ‘meaningfulness, comprehensibility and manageability’. These underpin a core construct of a ‘Sense of Coherence’ (SoC). Research taking a salutogenically oriented approach is especially important in maternity care systems, as most mothers and babies are healthy.^20^, ^21^ This study is, therefore, broadly based on the salutogenic philosophy, as opposed to being designed directly to measure the core concepts or the SoC.

The participants of the study were women who have given birth within 5 years of the date they complete the survey and who gave birth in Lithuania. The survey was run on an online platform (Survey Monkey®). It was hosted through an online portal (http://www.babiesbornbetter.org/surveyportal) and promoted online through social media (Facebook and Twitter), posts on COST Action member blogs and websites, discussion forums and maternity‐related websites in each participating country. Papers of Austria,^22^ Norway,^23^ Spain,^24^ Romania,^25^ Croatia,^26^ Iceland^27^ and Portugal^28^ have been produced on the B3 data, but none from Lithuania. To date, three waves of the survey have been completed, version one was open from February 2014 to December 2015.

Inclusion criteria for the current analysis were the indication of Lithuania as the country of residence, given birth within the last 5 years in Lithuania and provision of a minimum one answer to each of two of the open questions analysed in the current paper. The responses from 453 women were obtained and answers of 373 Lithuanian women responding to the B3 survey version one (from February 2014 to December 2015) who had given birth within the last 5 years were included in the current analysis. This is the largest survey to date reporting Lithuanian women's experiences of childbirth care. Ethics approval was obtained from the University of Central Lancashire, Built, Sport Health (BuSH) Ethics Committee (unique reference number BuSH 222).

### 2.2 Setting

In Lithuania, women can choose a governmental or private hospital for labour and birth. In both cases, care is provided by a physician‐led team which consists of obstetricians and midwives. Only some wards in a few hospitals provide midwife‐only‐led delivery care. In governmental settings, the payment for care is covered by insurance; however, families could pay for some additional services out of pocket. According to official statistical data, the total number of births in 2015 in Lithuania was 28608. Caesarean section was performed in 21.9% of all births (38.5%—elective, 61.5%—emergency caesarean). The rate of premature birth was 5.4%, and stillbirth—4.7%.^29^

Childbirth care at home was illegal during the survey period, and homebirths were not included in the official statistics. However, there was an active debate in society about changing the regulation around the place of birth. At the time of reporting on this survey, there is a government order describing home birth procedures, but no legal organisation has applied for a licence to provide this service. Despite being illegal, a small percentage of births do, in fact, take place at home. Thirteen women in our study reported homebirth.

### 2.3 Instruments

The B3 survey comprises 16 core questions, a comments box and two questions relating to information about the study results, and willingness to be contacted about future research. In the first (quantitative) part of the survey, information is collected on demographic characteristics, pregnancy problems, place of birth and the cadre(s) of staff who provided childbirth care. In the second (qualitative) part of the survey, women are asked to describe the childbirth care they received. Answers to the following open questions are involved in the current analysis: (1) What were the three best things about the care you got there? (2) If you had the power to make three changes in the care you had, what would the changes be?

### 2.4 Data analysis

The demographic and birthplace characteristics data were analysed with simple descriptives. The responses to open‐ended questions were analysed deductively, based on a coding framework created by the team who analysed the Austrian data of the B3 study.^22^ This framework includes three main categories regarding childbirth care experiences and views: (1) *service*, (2) *emotional experience*, and (3) *individually experienced care*, each further divided into subcategories (Table 1).

**Table 1:** Categories and subcategories of the coding framework.

Categories	Subcategories		
1. Service at the birthplace	1.1. Provision of services and rooms	1.1.1. Service 1.1.2. Infrastructure and rooms	
	1.2. Information and counselling	1.2.1. General information 1.2.2. Breastfeeding and baby care	
	1.3 Empowerment	
2. Emotional experience at the birthplace	2.1. Manageability	2.1.1. Positive 2.1.2. Negative	
	2.2. Meaningfulness	2.2.1. Bonding 2.2.2. Birth‐experience	
	2.3 Comprehensibility/feeling of safety		
	2.4. Companions	2.4.1. Partner 2.4.2. Others	
3. Individual experienced care at the birthplace	3.1. Self‐confidence	
	3.2. Caring/feedback of carer	3.2.1 Personality traits 3.2.2 Competence 3.2.3 Time/availability	
	3.3. Caring persons	3.3.1 Midwife 3.3.2 Physician 3.3.3 Others	
	3.4 Esteem	

A review of papers reporting childbirth experiences in Lithuania was performed before starting analysis; however, the review did not reveal any additional items to add to the a priori framework for data analysis. It was agreed that if any responses in the Lithuanian data set could not be allocated to the framework, they would be assumed to represent data specific to the Lithuanian setting and given a new code. Several new codes specific to Lithuania were derived after data analysis. Quotes for each theme are provided, to illustrate the meanings attributed to the themes by the respondents.

## 3 RESULTS

The mean age of participants was 31.9 (SD = 4.7) years. Forty‐five percent of participants had one child, 38.8% had two children and 16.4% of participants had three or more children. Gestational age of 37–41 weeks was reported in 87.1% of participants. Most of the respondents gave birth in the university hospitals and city hospitals in the two largest Lithuanian cities—Vilnius (*n* = 113) and Kaunas (*n* = 200). The third largest Lithuanian district, Klaipėda, was underrepresented.

**Figure 1:**
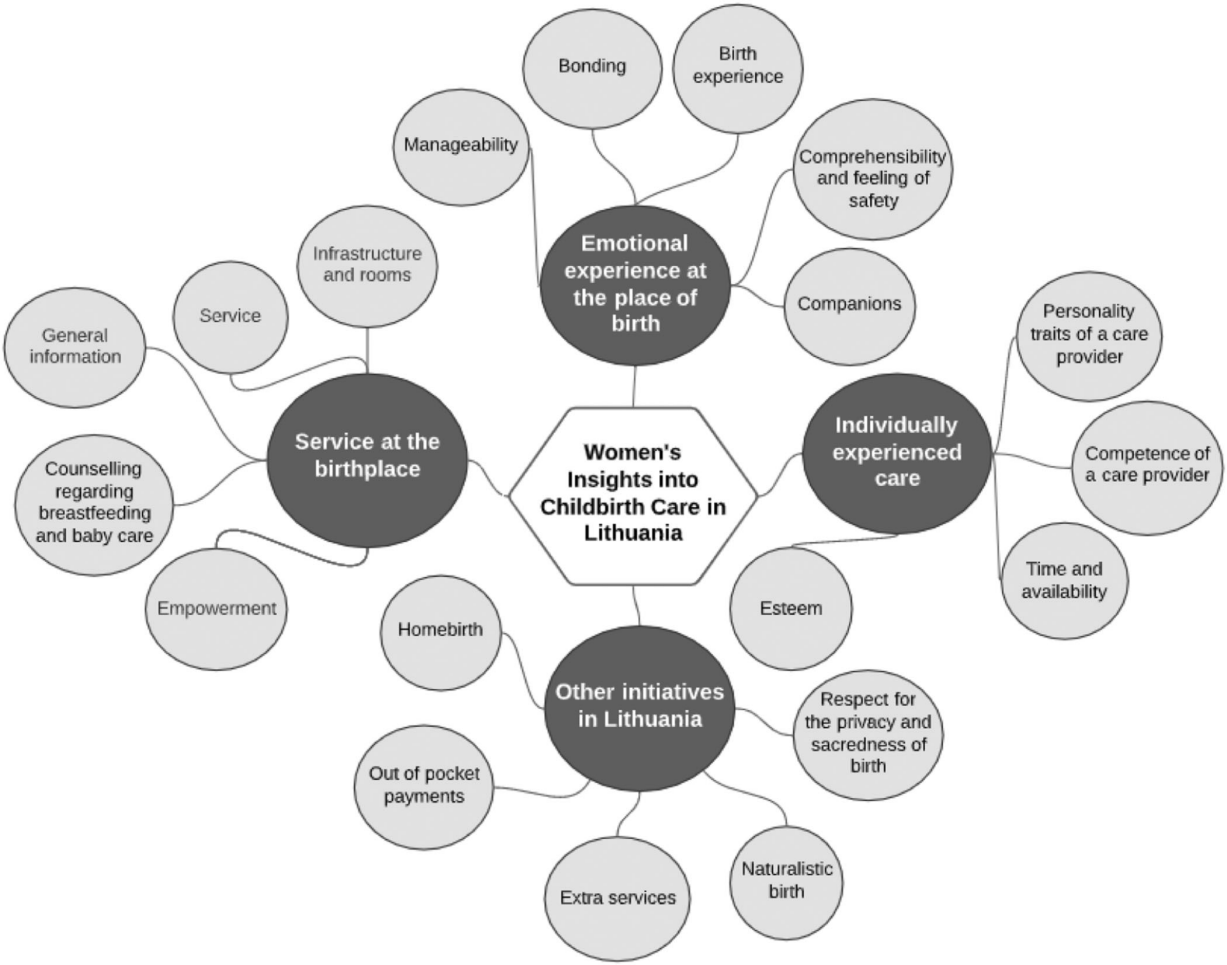
Map of categories and subcategories representing women's childbirth care experiences and views in Lithuania.

In total, 983 responses about the best things in childbirth care and 728 responses about aspects that could be improved were provided. The results are presented in a visual map of categories which reflects categories distinguished according to the axial category scheme relevant to Lithuanian data and additional categories specific to Lithuanian women's experiences in childbirth care (Figure 1).

Content analysis and counts of responses to open‐ended questions revealed that the most saturated category with the highest percentage of responses relating to the best aspects of childbirth care in Lithuania was *Individual experience of childbirth care* (49.5%). For the aspects that respondents would like to change, the most saturated category with the highest percentage of responses was *Service at birthplace* (37.9%).

### 3.1 Service at birthplace

Nearly one in four answers were related to the category of *Service at the place of birth* as part of the best aspects of care and more than one in three as aspects that women would like to change in childbirth care. Mothers talked about services and rooms, as well as needed information and counselling, especially about breastfeeding.

Women's voices highlighted a need for privacy and support for their autonomy. Privacy was a major part of the *infrastructure and rooms* responses to positive experiences. Women appreciated a comfortable and cosy individual or family room after birth. Usually, they had paid for this out of pocket, but in some cases private wards were free: ‘There was a separate free family ward after giving birth’. Some wrote about a comfortable, cosy and clean environment for giving birth: ‘They had spacious, light wards’. The special place for women and their partners without other families meant their better ability to take care of the baby and relax: ‘After birth, we were provided with a private, newly equipped ward, where the midwife left us in peace’.

Women also talked about desired changes in the provision of *service*. They wanted better space, equipment and kits in the wards where these services were lacking: ‘I wanted more equipment [to help me to have] a natural labour – chairs, balls…’. Lacking equipment created additional discomfort: ‘I needed to buy the epidural kit in the pharmacy while standing in the queue’. Women emphasised the lack of private, calm space in a healthcare institution: ‘I had a c‐section. There were a lot of women in delivery, so I spent a lot of time in the intensive care ward. Then they moved me into another ward and then into another…’.

Women highlighted good quality care as professional, attentive and careful clinical assessments (as well as medical interventions, if needed) for both mother and baby. They were satisfied with the continuous baby monitoring: ‘There was a constant following of the heart of the fetus’. Women also appreciated the responsiveness to their needs, for instance, the possibility of using alternative methods to help themselves during contractions: ‘They allowed me to use a ball in the delivery room’; ‘I had the possibility of using the shower during contractions of labour’.

On the other hand, the most saturated subcategory in its frequency and developed content reflecting women's views regarding needed changes in childbirth care was the lack of *empowerment*. Women wanted to be actively involved in decisions regarding services such as medical interventions: ‘I would not have chosen epidural analgesia if I was properly informed’. They wanted to feel included by staff: ‘I wanted an equal collaboration with the physician’. They also wanted to be informed about the processes: ‘I would like to know what intervention they are going to perform’. Though, often they did not receive proper information and communication: ‘I want to be informed and involved, not scared [by untrue information about risks] by staff<…>’. Women were also talking about respect for their autonomy to choose their birth plans, to have more time in the water during labour and be allowed to eat, and so forth. They also wanted to stay mobile and be allowed to choose their position for birth.

Information and counselling both regarding *general information* as well as *breastfeeding and baby care* was also remarked on. Women liked being invited and supported to breastfeed while being provided with practical information about the breastfeeding process. Providers' activities supporting breastfeeding were mostly highlighted as a positive experience: ‘After birth, midwives answered all my questions, and they supplied pyjamas, bedding, and mats when we needed them’; ‘There was fostering of breastfeeding (they did not suggest using formula)’. Though, some women were unhappy because they were advised to switch to formula, or they got poor support or advice about breastfeeding: ‘Staff had an attitude that it is normal for a first‐time mother not to know how to breastfeed, and to develop sore nipples’. They emphasised the need for more developed education about breastfeeding: ‘Give special attention to mother and baby in the first 24 hours, explaining to change a diaper and to feed the baby’.

### 3.2 Emotional experience at the place of birth

The codes with the highest number of allocated responses were *Comprehensibility and feeling of safety*; *Manageability* and *bonding* for both positive experiences as well as wanted changes.

For *comprehensibility and feeling of safety*, women described a general sense of emotional wellbeing, including a sense of security, and emotional support received as a reaction to attending personnel care: ‘There was a feeling of security during the whole process of labour and after it’. Positive experience included a sense that staff provided sincere care, reliability and a calming atmosphere, assuring women that everything was going to be alright: ‘I was sure that in case of necessity, necessary professional help would be provided’. Personnel's communication had also affected their wellbeing: ‘Distraction from the focus on pain – the midwife simply came to talk about other things than the labour’. To foster a greater *feeling of safety*, women raised issues that included the quantity and quality of examinations, and the need for more comprehensive care: ‘The woman should be followed during pregnancy and delivery by the same doctor. This should be obvious. The woman would feel safer’. Women also mentioned the lack of attention to mental health in general: ‘The physician could talk a little more about mental, not just physical issues after delivery’.

When reporting aspects of *manageability* women appreciated positive aspects of manageability such as efficiency and quick reaction when there was a need for help and decisions needed to be made during some problem or complication (e.g., bleeding after birth), when the staff offered direct support to help women give birth without interventions: ‘The midwife helped by holding my legs during the pushing stage and advising my husband on what relaxation exercises to do’; ‘I was shown how to decrease my pain during contractions’. Patient‐oriented support, not direct commanding was a positive experience and had a positive meaning for emotional experience. Women wanted to support and empowerment during difficult situations: ‘The midwife did not rush and was giving commands sparingly, she allowed the natural processes to take place (in my previous labours, excessive giving of commands made the labour only harder)’.

However, women also reported a lack of *manageability*, they complained about the overuse of medical intervention and various painful procedures, for instance, forcing them to lie on their back: ‘I was forced to lie on my back during delivery. If I wasn't, maybe I could avoid a perineal tear’. Some had fearful situations after hospital discharge which they believed could be avoided with better management so that they were not left alone with the problem. Others felt too much attention to bureaucracy and too little attention to women's voices and efficient management contributed to their suffering: ‘They were measuring contractions in a lying position. In that position contractions were weaker, so staff decided that I was not in labour. <…> they sent me to another hospital where my contractions became stronger. There nobody believed that I had contractions, and they told me to wait. I felt a huge disappointment because I needed to suffer all the contractions laying in the pregnancy ward where women had lunch or visitors’. Some women even felt humiliated by the staff: ‘I think the physician should not humiliate and frighten the woman in labour if she fails to perform<…>’.

Another important aspect was *bonding* experience. Positive comments related to *bonding* included skin to skin care, and keeping mother and baby together: ‘After giving birth we were left for a couple of hours to be alone together with the baby’; ‘After Caesarean section we were allowed to hold the baby on my chest for a while’. *Bonding* issues included the need to be supported during the bonding process. Women lacked the possibility to hold the newborn immediately on the mother's chest before any neonatal examinations are done and not be separated of baby: ‘I would prefer that the baby would immediately be put on the mother's chest, weight measurements can follow after’; ‘I wanted my baby to be left with me in the surgery room’.

Additionally, the role of *companions* was also connected to emotional experience during childbirth. This subcategory describes the importance of a partner's or other companion's presence and support: ‘It was very good that my husband could be there’. It was also important how healthcare workers accepted and treated the companions. Some participants mentioned the lack of the involvement of fathers (‘involving fathers as much as possible in the care of the newborn when the newborn is separated from the mother due to the illness’), as well as the need for them to be allowed to stay together for the first night.

### 3.3 Individually experienced care

One of the most elaborated categories describing positive experience was *Individually experienced care*, which was described by the feedback on specific characteristics of care providers behaviours, such as competence, personality traits, time or availability and encouragement of *esteem* in women in childbirth.

The *competence of the care provider* was the most frequent subcategory then describing positive childbirth‐care experience. Comments about care providers’ *competence* were largely focused on perceptions of professionalism and knowledge (‘The decisions taken by the doctors were the best for me and my child’; ‘There was advice on how to act correctly when, by myself, I didn't know how’), as well as the quality of teamwork and self‐organization (‘There was teamwork and self‐organization when complications arose’). Support of self‐confidence was also of great importance: ‘Experienced and qualified staff inspired optimism, self‐confidence’.

Women also emphasised their good experiences based on the *personality traits* of the care providers: being pleasant and friendly, caring, warm and sincere, tender and attentive. They reported that their best experiences included encountering the staff's willingness, smile and good sense of humour, helpfulness, respect, support, empathy, responsibility, discreetness, reliability, humanity, peacefulness, comprehensibility and flexibility. On the other hand, some women expressed the need for staff to be more friendly and more helpful: ‘Doctor did not talk with me, did not answer my questions and was angry’. In some cases they had experienced poor communication and negativity, including shouting at them, lack of understanding and respect, poor psychological support, and lack of humanity in general: ‘I would like a gentler doctor's tone while talking’; ‘I wanted higher competence and humanity from staff’.

In relation to *time and availability*, women expressed the importance of focused, regular attention from physicians: ‘After birth, midwives often came to the ward to ask whether I needed help or advice’. Concern and attention from care providers were mentioned the most often: ‘Personal care, as this was the only birth that day (no sense of conveyor as it happens in the large hospitals)’. Lack of *time and availability* of personnel was an issue in some cases. Some participants mentioned the lack of continuity of care after leaving the delivery ward or at home after discharge from hospital: ‘I wanted to have a midwife's visit at home’. They described frequent shift changes in staff, the sense of rush or even unavailability of care providers: ‘I wanted to have less personnel changes after delivery. Every time I saw a new midwife, I needed to explain everything once again’; ‘I wanted to have a more accessible physician’. It bothered and strengthened the feeling of insecurity. Furthermore, mothers expressed the need for stability and constant care by practitioners they already knew.

Women also talked about the encouragement of mothers' *esteem*, which included staff's consideration of women's needs, wishes and requests during and after labour: ‘I could just be with my husband; the midwife was calmly entering only when it was needed’. Leaving the personal space for women and their close ones was one of the verbalised needs: ‘There was minimal interference of personal space’; ‘They did not interfere in the process, they just observed it’. Otherwise, lack of esteem in some cases included lack of respect for women's wishes, negative comments in their presence, being ignored, and being exposed to too many personnel member: ‘I wanted to rest a little bit between the contractions, but the physician instructed me to move’. In some cases women felt ignored and depersonalised: ‘Patient's [my] wishes were ignored, I felt contempt [from staff]’; ‘Women in delivery – [need to be treated as] humans, not only as meat and physiology’.

Some women also reported *caring persons* as an important part of childbirth experiences without providing more detailed information regarding the exact behavioural patterns or personal characteristics of these persons.

### 3.4 Other initiatives

Some of the answers did not fit any category of the applied deductive framework. Part of the answers was specific to particular healthcare policy situations, including the current Lithuanian movement of women for individualised natural humanised care. One of the topics was *birth care at home*. Some women reported as a positive aspect of childbirth care the fact that they had home deliveries or had a chance to be supervised by an obstetrician who provided birth care at home: *‘*I had a home birth*’*.

Over the time period covered by the survey, as noted in the introduction to this paper, it was illegal to have a home birthcare in Lithuania, and extensive debates were taking place in society about possible changes to this regulation. However, all discussions of reasons for home birth showed a lack of empowerment and respect for autonomy in hospital settings. When indicating preferable changes women mentioned willingness to have safe and legal birth service at home: ‘I would prefer that more medical personnel be prepared to deliver birth service at home and women could have a choice’.

Another topic of women's answers was *out‐of‐pocket payments*. Women indicated that they enjoyed personalised care at the hospital, but they paid it out of pocket. This practice was also poorly regulated and out‐of‐pocket direct payment was illegal. Despite the fact that professionalism did not differ between the payment and nonpayment cases, women preferred to do this to ensure their autonomy during the birth process: ‘The doctor was amazing, but I had an agreement about birth care with him’; ‘I had paid to the anaesthesiologist; she was very kind’. *Out‐of‐pocket payments* were mentioned as negative aspect of childbirth care. Women advocated for higher salaries for personnel or regret too expensive payments both for personnel or for advanced service in the hospital. Some of them felt forced to provide out‐of‐pocket payments, and some of them were afraid not to suggest these payments expecting a lower quality of service. Some of them sided with the movement not to support out‐of‐pocket payment practices: ‘I don't think women should have to give “envelopes” [bribe, illegal payment]’.

Amongst positive aspects of childbirth care *extra services* were mentioned, which were a deviation from normal care, such as a possibility to be discharged earlier, some gifts, and so forth: ‘Early discharged home’; ‘The baby was watched another six days after birth, although Apgar score was 9 points’.

Some women positively commented that they did not experience some of the negative things which had been discussed in the media. They were happy to have a more *naturalistic birth* and postpartum care approach, more personalised attention after delivery or even less hospital personnel in attendance than they expected, but more of their husbands’ attention during labour, avoidance of moralisation and disrespectful behaviour and even the provision of small talk for creating comfort: ‘It was night and the doctor was sleeping – we were calmer’; ‘Very caring and non‐moralising environment’.

Women also wanted more *respect for the privacy and sacredness of birth*, quiet discussion amongst personnel, possibility not to be interrupted by others in birth: *‘*I would like not to hear the screaming of other women in birth’; ‘The nurses of the intensive care ward could discuss more quietly’.

## 4 DISCUSSION

In line with salutogenic principles, the findings of this study capture what went well for respondents, as well as what could be improved. Specifically, in the domain of emotional experience, the core principles of salutogenesis (meaningfulness, manageability and comprehensibility) were directly reflected in the data. More generally, the findings show that women value both clinical and psychosocial aspects of intrapartum care, as demonstrated in a recent systematic qualitative review of studies reporting what matters to women around the world.^7^ We interpreted similar areas of responsibility as good examples if provided and bad examples if reported to be lacking. The implementation of these aspects which were missing would not require new technologies or investments, but additional services, and improved emotional and intrapersonal aspects of care. Issues that matter to women in Lithuania are multidimensional and similar to those reported in other countries where the B3 qualitative responses have been analysed, particularly positive characteristics and feedback of care providers,^22^, ^26^, ^27^ respect and choise^28^ and possibility to have companions.^23^

Despite the fact that some women reported paternalistic attitude‐based practice, others appreciated a shift towards patient‐oriented care. This suggests that the Lithuanian healthcare system is in transition towards improvement. This trend is reinforced in the findings of more recent surveys.^30^

Our study revealed that a sense of empowerment in maternity care is an important expectation of women. This is beneficial to maternal wellbeing. Both empowerment and a sense of control have been reported as a central need during childbirth^31^ and as one of the main aspects of a positive experience of childbirth.^15^, ^32^ Lack of a sense of empowerment was a notable dissatisfaction factor.^33^ In support of this finding, a systematic review of 27 articles showed that interventions supporting empowerment are associated with reduced perinatal depressive symptoms and rates of prematurity/low birth weight.^34^

Being in a comfortable environment was also highly valued. Many women responding to the first wave of the B3 survey were only able to access such settings by paying extra for them. However, as more public hospitals in Lithuania improve their environments, this should be available to more women through standard governmental insurance. The added value of the emotional security and sense of privacy provided by improved birth settings should not be overlooked.

Communication that enhanced empowerment, psychological support and respect was highly valued. Violation of dignity through poor communication was evaluated very negatively. According to other studies, ineffective communication, lack of supportive care and loss of autonomy can be understood as examples of disrespect in childbirth care,^35^ thus creating tension between provider and patient.

Partner participation was mentioned as part of emotional support. Husband support was important for mothers in previous studies. Positive attitude and the necessity for spouse support during delivery have been described in Lithuania, as the participation was strengthening the relationship between the wife and the newborn, relieved pain of childbirth, reduced anxiety and provided confidence.^36^ In our study, the partner support was not verbalised possibly due to the understanding that spouse participation is a natural process and more painful topics such as empowerment or provision of care were highlighted.

Out‐of‐pocket payments and exclusive, additional services are part of healthcare in many other post‐Soviet countries.^37^ This is partly illustrated in the three aspects that did not fit the a priori theoretical frame; namely the debate on home birth legislation; the need for bribes to obtain some aspects of standard care; and miscommunication on issues that were important for the mother, which they felt as scaremongering, rather than as collaborative encouragement.

The home birth issue has been discussed above. The issue of bribes for improved services is more nuanced. In Soviet times, the political will was to keep salaries for physicians low, ‘because the folk will feed them’. This created the tradition of out‐of‐pocket payment for health services or even feeling in the society of a need to pay out of pocket even if it was not asked for. Even though these activities are now illegal, there is a broad discussion in Lithuania about continued willingness to give and willing to receive these payments. Society was in transition on that issue during the study and we discovered a tension and dilemma amongst young mothers—is a service better because of payment; or is it worse because of nonpayment; and do I feel I want to pay when the service was good because in this way I want to show appreciation?

As the main care aspects which were discussed in the Lithuanian sample were related to services delivered by healthcare personnel, such as psychological support, empowerment, communication and illegal payments are reconstructed as a tool to increase the probability of enhanced care. This can create social tension between the providers and women, even though personal attitudes towards the ethics of these payments differ across the population.^38^

### 4.1 Limitations

Online surveys can attract more proactive women than those in the general population. In addition, distribution using parental forums could have engaged more respondents who are inclined to be active change agents. This could also be seen as a strength, however, as women in such groups are often addressing issues that matter to many others who remain silent, and so their contributions can allow important issues to surface. Another limitation is that a limited number of sociodemographic characteristics were collected in the survey. It is possible that participants were more educated, and more motivated to report suggestions for changes in the maternity care system than the general population, as this is often the case for survey responders.^39^

## 5 CONCLUSIONS

The findings of this study suggest that the Lithuanian healthcare system is in transition from prior paternalistic attitude‐based practices to a shift towards patient‐oriented care. Implementation of improvements suggested by Lithuanian respondents to the B3 survey would require some additional services, improved emotional and intrapersonal aspects of care and a more active role for women. Further research could entail the use of the geomapping capacity of the survey to locate hospitals where women only report good care, and to undertake a more in‐depth study, including interviews with staff and women, to establish what they are doing well, in line with salutogenic principles.

## AUTHOR CONTRIBUTIONS

*Study design, supervision, review and editing*: Giedrė Širvinskienė. *Qualitative data analysis and interpretation*: Švitrigailė Grincevičienė. *Qualitative data analysis and interpretation*: Rasa Pranskevičiūtė‐Amoson. *Introduction section preparation, review and editing*: Milda Kukulskienė. *Study design, review and editing*: Soo Downe. All authors critically revised the manuscript. All authors gave final approval of the version to be published and agreed to be accountable for the work.

## CONFLICT OF INTEREST STATEMENT

The authors declare no conflict of interest.

## ETHICS STATEMENT

Ethics approval was obtained from the University of Central Lancashire, Built, Sport Health (BuSH) Ethics Committee (unique reference number BuSH 222).

## Data Availability

The data that support the findings of this study (in Lithuanian) are available on request from the corresponding author. The data are not publicly available due to privacy.
